# Interparental violence: child emotional awareness, protective factors, and symptom profiles in a comparative analysis

**DOI:** 10.3389/fpsyt.2025.1418332

**Published:** 2025-06-19

**Authors:** Eva M. Ortiz Jiménez, Juana Gómez -Benito, Selene Liz Llach, María Lamarca, Susana Ochoa

**Affiliations:** ^1^ Department of Child and Adolescent Mental Health, Hospital Sant Joan de Déu, Esplugues de Llobregat, Spain; ^2^ Department of Clinical and Health Psychology, University of Barcelona, Barcelona, Spain; ^3^ Department of Social Psychology and Quantitative Psychology, University of Barcelona, Barcelona, Spain; ^4^ Group on Measurement Invariance and Analysis of Change (GEIMAC), Institute of Neurosciences, University of Barcelona, Barcelona, Spain; ^5^ Grup MERITT, Fundació Sant Joan de Déu, Institut de Recerca Sant Joan de Déu, Esplugues de Llobregat, Barcelona, Spain; ^6^ Departament de Psicologia Clínica i de la Salut, Facultat de Psicologia, Universitat Autònoma de Barcelona, Cerdanyola del Vallès, Barcelona, Spain; ^7^ Parc Sanitari Sant Joan de Déu, Sant Boi de Llobregat, MERITT Group, Sant Joan de Déu Research Institute, Barcelona, Spain; ^8^ Centro de Investigación Biomédica en Red de Salud Mental (CIBERSAM), Madrid, Spain

**Keywords:** emotional awareness, child abuse, exposure to intimate partner violence, symptoms, protective factors

## Abstract

**Objective:**

This study aimed to analyze the differences between children exposed to interparental violence (EIPV) and non-EIPV children aged 8-12, in a) Emotional awareness (EA), b) Protective factors of resilience (external and internal) c) Externalizing/internalizing symptoms, somatic complaints and moods.

**Method:**

A descriptive design study was conducted with a total of 115 participants (60 boys and 55 girls) from three Child and Adolescent Mental Health Centers in the metropolitan area of Barcelona, Spain. Student’s t-test was used to compare the EIPV and non-EIPV groups, and logistic regression models were employed to identify the most relevant factors associated with EIPV.

**Results:**

EIPV children exhibited significantly lower scores in differentiating emotions (DIE) and analyzing one’s own emotions (ANE) compared to non-EIPV children. Additionally, EIPV children demonstrated more social skills problems, lower loneliness/social anxiety, lower resilience, mainly in empathy and internal protective factors, autonomy, and reduced self-esteem. They also reported higher levels of externalizing and internalizing symptoms, somatic complaints, and negative mood states such as fear, sadness, and anger. The variables that explained group membership in EIPV were DIE, ANE, and total externalizing symptoms.

**Conclusions:**

This study provides valuable insights into the role of EA, symptoms, and protective factors in EIPV children in a clinical sample. Lower EA, fewer protective factors, and higher levels of internalizing and externalizing symptoms in EIPV children when compared to their non-EIPV counterparts. Including a structures evaluation of EA and Protective Factors in the EIPV child population would improve diagnostic accuracy of trauma and the design of specific psychotherapies interventions aimed at reducing symptoms and promoting mental well-being in children aged 8 to 12 exposed to IPV.

## 1 Introduction

### 1.1 Intimate partner violence

In recent years, the issue of intimate partner violence (IPV) has gained significant attention. In Spain, it has been reported that 14% of women over the age of 16 have experienced some form of violence from a current or previous partner within the past four years (1). Additionally, approximately 19% of individuals seeking assistance from childhood mental health centers have been exposed to IPV (2). These statistics highlight the prevalence and impact of IPV on individuals and the urgent need for intervention and support for those affected.

Edleson et al. (3) described exposure to interparental violence (EIPV) as multiple experiences in children who live in households where adults use violence as a form of coercion against their partners, which may include forcing the child to observe an attack on the mother, using the child as a spy, witnessing the consequences following a traumatic incident, seeing their mother injured, police intervention, and transfer to a shelter. Furthermore, Holden (4) demonstrated that the subsequent consequences of the incident also had a traumatic effect on these children.

This study aligns with the theoretical framework of developmental theories, attachment theories, and resilience theories. Developmental theories considers that consequences in children EIPV depend on the interaction produced between the characteristics of the violence, the surrounding context’s capacity to provide support, and the child’s developmental coping abilities (emotional, cognitive, and behavioral development, among others) in shaping the consequences of EIPV (5, 6).

Drawing from research in attachment theory (7), interparental conflict is perceived by children as a threat to their sense of security in the parental relationship. As a result, these patterns of response, which include increased anxiety and fear of conflict, intense avoidance of conflict, and active participation in attempts to mediate or distract parents from their disputes, may be replicated in other interpersonal relationships.

Finally, resilience has emerged as a key interest in recent years. A heightened focus has been placed on identifying factors and mapping developmental trajectories associated with resilient functioning in EIPV youth (8, 9), thus aligning the field with the strengths-based framework that defines direct practice models (10).

### 1.2 Emotional awareness: concept and factors

Emotional awareness (EA) is the ability to be conscious of one’s own emotions and those of others, as well as to perceive the emotional climate of a setting (11). Children are taught to find a balance between their own desires and the demands of society, without jeopardizing their social relationships. The process of socialization in normal development leads to the control of emotions, attention, and insight into one’s own emotional responses in EA, and it is considered a prerequisite for effective emotional regulation (12). It is a basic competence that allows for the development of the rest of social skills (13).

It has been established that EA has 6 components (14, 15): a) differentiating emotions (DIE), b) verbally sharing of emotions (VSE)—the ability to speak about emotions, c) not hiding emotions (NHE)—the tendency to express one’s own emotions frankly and openly, d) bodily awareness of emotions (BAE)—the ability to understand the nexus between emotional activation and body symptoms, e) attending to the emotions of others (AOE), and f) analyzing one’s own emotions (ANE).

Some constructs are closely related to EA, such as emotional intelligence (EI) and alexithymia. EI is a broader concept in which EA is included. The EI skills model conceptualizes EI through four basic skills: the ability to perceive, evaluate and express emotions; the ability to access and/or generate feelings that facilitate thought; the ability to understand emotions and the ability to regulate emotions (16). Alexithymia, on the other hand, is more restrictive, as it refers to problems identifying and describing one’s own feelings (17), rather than including analyzing others’ emotions.

Previous studies suggest that in children aged 8 to 12, EA significantly influences their development, including health, social competence, and academic and social adaptation. This ability is directly linked to how they handle negative emotions and their capability to distinguish between different emotions, impacting their physical and emotional well-being. Additionally, it’s observed that by age 9, children become more skilled at reflecting on their own emotions and understanding the rules for hiding emotions (18).

A corpus of literature has highlighted the importance of EA during childhood and adolescence, finding a relationship between high EA and low aggressiveness (19). Conversely, low EA is associated with internalizing problems (12, 13), psychosomatic disorders (20), behaviour problems (21), worsened emotional regulation (22, 23), social maladjustment (24), higher suicidal ideation and suicidal attempts, as well as Post-Traumatic Stress Disorder (PTSD) symptoms in adolescents (25).

### 1.3 EA and child exposed to IPV

Children exposed to violence and other forms of adversity may struggle with recognizing and differentiating emotions in facial expressions (26), as well as labeling and identifying emotions (27, 28); these difficulties may be attributed to stress-related disruptions in the detection of internal bodily states (29).

The presence of alexithymia symptoms may also be linked to developmental trauma exposure. According to developmental models, cognitive abilities related to EA and expression undergo natural developmental progression within the context of attachment relationships. However, traumatic experiences and neglect during childhood can disrupt this development, potentially leading to more severe cases of alexithymia in adulthood (30).

EA in EIPV children remains a relatively under-researched area, though several studies have started shedding light on this topic. Goldsmith and Freyd (31) conducted research specifically on children exposed to emotional abuse and found that they exhibited lower levels of emotional competence and experienced difficulties in identifying their emotions compared to non-abused children. Katz et al. (32) found that decreased EA was associated with higher levels of internalizing and externalizing symptoms in young children and preadolescents in a community population, indicating its relevance for psychological well-being. Recently, in a study about children exposed to IPV, Ortiz et al. (33) showed that lower EA was associated with total externalizing and internalizing symptoms and more somatic complaints. Greater EA was linked to improved social skills, higher self-esteem, and both external and internal factors of resilience.

### 1.4 Resilience and protective factors and child exposed to IPV

Although the term resilience is used inconsistently in popular media, there is considerable consensus among leading resilience researchers, often defining it as the process of adapting well in the face of adversity, trauma, tragedy, threats, or significant sources of stress. This can involve resilience in families, communities, and systems that impact children’s development (34).

Protective factors, according to Masten et al. (34, 35), include a range of conditions or attributes in individuals, families, communities, or the larger society that, when present, mitigate or eliminate risk in families and communities, thereby increasing the health and well-being of children and families. These factors might include traits like self-regulation, positive relationships with caring adults, intelligence, and skills in problem-solving, among others.

Recent reviews emphasize positive parent-child bonds and self-regulation as key resilience elements against violence (36, 37). A 2019 meta-analysis found that self-regulation, family, school, and peer support mitigate the impact of violence, including IPV (38). Cognitive, emotional, and behavioral responses to adult conflict also protect children (39), moderating the effects of parental strife on child adjustment (40). Moreover, conflict and IPV can lead to dysfunctional parenting and fewer supportive, accepting, and affectionate interactions with children (41, 42).

### 1.5 Child exposed to IPV and symptomatology

In the last 50 years there has been a flurry of studies on the effects of IPV exposure on children. Exposure in children was linked to a higher risk of behavioral, emotional, social, and cognitive difficulties, and the impact of IPV on those outcomes varied based on a child’s age and developmental stage (10). Many studies demonstrate that children EIPV have worsened mental health, and more affective, behavioral, and cognitive symptoms, in comparison to those not exposed to abuse (43, 44), as well as greater problems with social and interpersonal skills (45), greater parent-child conflict (46) and increased hostility with siblings (47).

Additionally, research has shown that children EIPV are at considerable risk of experiencing physical health complaints, irrespective of whether they have also been victims of other forms of abuse; child witnesses of IPV more frequently experienced health complaints, especially related to eating, sleeping, pain problems, and self-harm (48).

Finally, some studies have argued that children EIPV experience multiple traumatic situations over an extended period, aligning more closely with the concept of Complex PTSD (CPTSD) beyond the diagnosis of PTSD (49, 50). Additionally, recent studies have demonstrated that interventions targeting emotional competencies and interpersonal relationships effectively reduce PTSD symptoms in children EIPV (51).

This study seeks to enhance understanding by merging developmental theories and resilience models within an ecological framework, focusing on children EIPV. It examines how such experiences influence children’s emotional and social development across various environments, including family, school, and peer interactions. Key aspects of investigation include internal competencies like EA, social skills, and self-esteem, along with the external and internal PF that contribute to resilience. The study also considers the broader implications of attachment theories, particularly how exposure to IPV might lead to disorganized attachment patterns, affecting the children’s ability to form secure emotional bonds.

While the existing literature provides valuable insights into the negative consequences of EIPV on children’s well-being, several gaps remain. Studies often focus on broad symptomatology rather than the specific mechanisms underlying these effects, leaving questions about how EA develops in EIPV children and its role as a protective factor (51). Additionally, most research relies on general population samples, with limited attention to clinically referred children, who may experience more severe symptoms and require targeted interventions. Although age-related differences in IPV impact have been acknowledged (10), little is known about how these effects manifest in middle childhood (8–12 years), a critical yet underexplored stage. Further, inconsistencies in defining protective factors—some studies emphasizing social skills and self-esteem (45), while others focus on family dynamics and interpersonal functioning (46)—complicate efforts to identify the most effective buffers against IPV’s impact.

Although EA in children exposed to EIPV has begun to be studied, it remains a relatively unexplored area. There is still much that is not understood about how EIPV specifically affects EA factors, psychological symptoms, somatic complaints and protective factors in children aged 8–12 years in a clinical sample. Furthermore, there are no studies comparing EA, protective factors and symptoms in a clinical population of children aged 8–12 with EIPV compared to children without exposure in the same age group.

This comparative study addresses this gap by: a) examining how exposure to IPV affects this relationship and which specific protective factors moderate this effect in children EIPV; b) identifying specific protective factors that mitigate the negative effects of exposure to violence, as it is crucial for the development of intervention and support programs aimed at children at risk; c) enriching the existing literature, providing a more nuanced understanding of the consequences of EIPV on children’s EA and psychological well-being. Additionally, the findings could have direct implications for clinical practice, informing the development of more effective intervention strategies to support children exposed to IPV.

Our interest in 8-12-year-olds, often termed the “forgotten years”, stems from the scarcity of research on this developmental period compared to early childhood and adolescence. Despite being a critical stage for cognitive, social, emotional, and physical advancements, middle childhood has received less attention in psychological and developmental research (52, 53). During this phase, children enhance their ability to regulate emotions, develop complex social skills, and establish a sense of identity and self-efficacy, all of which are foundational for later well-being. Moreover, their growing independence, peer relationships, and academic challenges make this a sensitive period where exposure to adverse experiences, such as IPV, may have a profound impact on their emotional and psychological adjustment.

However, most research on IPV exposure has focused on early childhood attachment disruptions or the long-term consequences in adolescence, leaving a significant gap in understanding how IPV affects children in this intermediate stage. Given that this period represents a window of opportunity for intervention.

This study aims to analyze the differences between EIPV and non-EIPV children aged 8-12, in a clinical sample: a) comparing the level of EA between children who have been EIPV and those who have not; b) assessing differences in protective factors, including external protective factors (family support, social support, and community support networks) and internal protective factors (personal abilities and resources such as self-efficacy, self-esteem, purpose and meaning in life, problem avoidance, and coping skills), as well as personal resilience factors (PRF) (autonomy, humor, creativity, empathy, social skills, and self-esteem); c) comparing internalizing symptoms, which are directed inward and affect the child’s emotional world (e.g., anxiety, depression, social withdrawal, and somatization), and externalizing symptoms, which manifest outwardly through disruptive behaviors, aggression, impulsivity, disobedience, and difficulties in anger control; and d) determining which factors of EA, symptoms and protective factors are most useful to explain EIPV and non-EIPV children.

The hypotheses of this study are as follows; a) There is a lower capacity for EA in children aged 8–12 who have been exposed to IPV compared to those who have not been exposed; b) EIPV children have fewer protective factors, including external and internal protective factors of resilience, social skills, self-esteem and; c) EIPV children experience more severe somatic complaints, internalizing and externalizing symptoms compared to non-EIPV children; d) Variables related to EA, symptoms, and protective factors can be used to discriminate between EIPV and non-EIPV children.

## 2 Materials and methods

### 2.1 Design

This is a multicenter, cross-sectional comparative study.

### 2.2 Participants

The participants were drawn from three Child and Adolescent Mental Health Centers (CSMIJ) Sant Joan de Deu Hospital in the Barcelona metropolitan area in Spain: Cornellà, Mollet del Vallès and Vilafranca del Penedès. There was a medium-low socioeconomic level (54). The participants exposed to IPV in this study were enrolled in the Testigos de Violencia Doméstica (TEVI) program, [Witnesses to Domestic Violence from the Child Mental Health Center], which aims to identify instances of domestic violence and provide therapeutic interventions to individuals affected by it. The TEVI program consists of a multidisciplinary team of psychologists, psychiatrists, and social workers who collaborate with various community services, including educational, social, medical, and adult programs for domestic violence. Included in the study were EIPV and non-EIPV patients between 8 and 12 years old. Exclusion criteria were patients with a diagnosis of autism spectrum disorder (ASD), those with an intellectual handicap, as well as those with active psychotic symptomatology.

Data collection occurred between 2021 and 2023, and certain potential confounding factors, such as socioeconomic status, were not collected. These have been acknowledged in the limitations section of this study.

To calculate sample size we took into account the percentage of EIPV children who attended community centers and came up with a sample of 50, given that it would be possible to detect correlations of r=.33 with a significance level of 5% and a power of 80%. This effect size was calculated to study the relationship of variables within the group of EIPV children and to facilitate comparison, the same number of non-EIPV children, 50, was included. However, considering the lengthy nature of the evaluation and the possibility that some children might drop out during the process, we included an additional 20% of the sample to compensate for potential losses in the evaluation.

Among a total of 119 subjects, 115 subjects were included, 58 subjects who had been exposed to IPV and 57 who had not been exposed to IPV, all between 8 and 12 years old. A total of four subjects were excluded, one due to another type of violence between siblings and parents, two due to diagnostic ASD, and one due to intellectual handicap.

The selection of the children included in the EIPV group was carried out through two scales on violence: the children received the Conflict Properties Scale of the Children’s Perception of Interparental Conflict (CPIC) Questionnaire, with a cut-off point equal to or greater than 13 for inclusion, or the mothers received the Woman Abuse Screening Tool (WAST) with a cut-off point greater than 14 and their children had scored over 8 in the CPIC for inclusion. We took into account previous studies suggesting low agreement between informants when reports come only from the mothers of exposed children, and the importance of obtaining information directly from the exposed children themselves (55, 56).

### 2.3 Procedure

Recruitment of the EIPV children group was carried out by CSMIJ therapists. They called for informational sessions in their centers, explained the aims and procedures of the study, and made a call for participation.

The request for participation in the study was made to the legal representatives of the children (parent or guardian) while children provide assent, and to those 12-year-olds who fulfilled the inclusion criteria.

The study was designed just before COVID-19 pandemic, but was carried out during it and as such, it was necessary to adapt the recruitment method through online and telephone communication. The information sheets and questionnaires were adapted to the REDCap software in order to be administered online during confinement.

The parents or guardians of the children were contacted in person or by telephone by the principal investigator of the study in order to request their participation in it. Afterwards they were sent a link, by email, to the information sheet and the informed consent form to be signed. Following this, questionnaires were sent to the parents/guardians and the children. Families were contacted by telephone to request their attendance with the children for an assessment.

### 2.4 Instruments

Assessments were carried out with the parents and the children. The total time for assessment was 1.5 hours, in two 45-minute sessions. A therapist accompanied the children and explained how to complete the questionnaire. Details of the instruments follow.

#### 2.4.1 Sociodemographic questionnaire

A series of data related to sex, age, ethnic group, education level, parents’ profession, parents’ marital status, and family circumstances. In addition, there were also questions concerning first degree relatives with mental illness and mental diagnosis (according to DSM-5; 57) based on clinicians.

#### 2.4.2 Intimate partner violence assessment

WAST – Woman Abuse Screening Tool (58, 59). This is a screening instrument for the presence of violence in the couple, designed to be administered by primary care physicians. It consists of 8 items with three response choices on a Likert-type scale from 1 to 3, geared to exploring first and foremost the presence of tension and fear in the couple, and then including direct questions on episodes of emotional, physical, and sexual violence. The total score of the scale (8-24) is obtained by adding the proportions corresponding to the 8 items. It has a sensitivity rating of 94.5% and specificity of 90.5%, and an alpha coefficient of.95. It was used to confirm the presence of interparental violence, with a cutoff point of 14 based on Garcia-Esteve et al. (59).

CPIC – Children’s perception of interparental conflict scale (60, 61). This self-report questionnaire evaluated the children’s perspectives on the conflicts that occur between their parents. The original scale consists of 49 items, but we applied the short version of 36 items (62) which can be applied to children between 9 and 12 years of age. The questionnaire is organized into three dimensions: conflict properties, guilt and perceived threat. This abbreviated version of the CPIC has shown high overall reliability with a Cronbach’s alpha of.91 for the total scale, and values ranging from.77 to.82.

#### 2.4.3 Emotional awareness assessment

EAQ 30 – Emotional Awareness Questionnaire (14, 63). This tool evaluates the ability to differentiate emotions, express them verbally, not hide them, be aware of one’s body, analyze one’s emotions, and attend to the emotions of others. It includes 30 items with a Likert-type scale of three anchors of response (1 = never, 2 = sometimes, 3 = often). Which can be applied to children between 8 and 12 years of age. The factors show acceptable reliability indices (between α=.63 and α=.68).

#### 2.4.4 Symptoms assessment

CBCL – The Child Behaviour Checklist, 6 to 18 years of age (64, 65). It is made up of 113 items, assessed with a Likert-type scale with three response options: false or rarely, sometimes, and true or almost always. The scale provides a profile of the subject in two large areas of behavior: internalizing and externalizing. It has a Cronbach’s alpha of.97.

CDI – The Children’s Depression Inventory Kovacs (66). This is a self-administered scale that evaluates depression in children and adolescents aged 7 to 17. It encompasses areas related to depressed mood, interpersonal problems, feelings of incapacity, anhedonia, and low and negative self-esteem. It contains 27 items. For the community sample, the internal consistency (Cronbach’s Alpha) ranges from.82 at the test time to.84 at the retest. In the clinical sample, Cronbach’s Alpha reaches a value of.85.

STAIC – State - Trait Anxiety Inventory for Children (67). This questionnaire, designed to measure anxiety, can be used with children from 9 to 15 years of age. It is made up of two scales with 20 items each. The ‘anxiety state’ scale assesses transitory anxious states while the ‘anxiety feature’ scale measures the relatively stable differences in propensity to anxiety in children. It has acceptable reliability with a Cronbach’s alpha of.70.

SCL –. Somatic Complaint List (68). Self-reported scale designed for children 8 years and older, and made up of 11 items, the SCL asks participants to rate the frequency with which they experience certain bodily complaints, such as a stomachache, on a three-point scale (1 = never, 2= sometimes, 3= often), yielding a single overall score for frequency. Its reliability was high, with a Cronbach’s alpha of.84.

MOOD – Questionnaire on Moods (69). Self-reported scale designed for children 8 years and older. Assesses the frequency of four mood states: fear, sadness, happiness, and anger, during the preceding four weeks. It is made up of 20 items, with a Cronbach’s alpha of.81.

#### 2.4.5 Protective factors assessment

Resilience Inventory for Children (70). This is an inventory of individual resilience features that measures 5 personal factors: self-esteem, empathy, autonomy, humor, and creativity. The inventory has 48 questions, framed both negatively and positively. It was designed for use in children aged 7 to 12.

GA-RE14 – Resilience Scale for Mexican Children (71). This scale assesses the capacity of the individual to adjust and adapt “after having been subjected to adversity” (72, p. 111). It is designed for use with school-age children. It includes 14 items with 5 Likert-type responses. It measures 3 factors: internal protective factor (IPF), external protective factor (EPF), and empathy factor (EF), with an overall Cronbach’s alpha of.86.

MESSY – The Matson Evaluation of Social Skills with Youngsters (73, 74). This measures the specific social skills involved in adaptive and non-adaptive behavior. It is designed for use with school-age children and adolescents. It includes 62 items assessed with a 4-point Likert-type scale. It has five factors: aggressivity, appropriate social skills, friendship, conceit, and loneliness. The reliability of the instrument was adequate (α = .81).

RSES – Rosenberg Self-Esteem Scale (75, 76). This is one of the most widely used instruments to measure self-esteem. Initially it was used with children over the age of 11, but now its use has been extended to other age groups as well. The scale measures a single factor of global self-esteem through 10 items on a four-point Likert scale, where responses range from 1 (Strongly disagree) to 4 (Strongly agree), both positively and negatively worded items—5 of each. The reported reliability (α = .75) indicates adequate internal consistency.

### 2.5 Ethical considerations

Both the parents/legal guardians and the children signed informed consent for participation in the study. The research project, approved by the Ethics Committee of Sant Joan de Déu (reference: PIC-187-20), was based on the principles for medical research in human beings enshrined in the Helsinki Declaration (1964), and its more recent revision in Fortaleza, Brazil (77).

### 2.6 Statistical analysis

A descriptive analysis was made of the sample. We applied the Student’s t test to compare both groups (EIPV and non-EIPV children) in variables with a Gaussian distribution, and Mann-Whitney U in the variables in which there was no normality in the distributions. Statistical significance was set to.05. The Cohen’s d coefficient was used as the effect size measure for comparisons made with the student’s t-test, and the r coefficient was used for comparisons made with the Mann-Whitney U test.

We did not carry out multiple comparison corrections owing to the exploratory nature of the study (78).

Three stepwise logistic regression analyses were conducted: one with emotional awareness variables, the second one with protective factors variables and the third with symptoms variables. After that, we conducted a hierarchical logistic regression including the previous significant variables. In the first block we include emotional awareness variables, in the second block we include protective factor variables and in the third block we included symptoms variables The statistical package SPSS (version 25.0) was used.

Data was not preprocessed for the analysis, as most of the patients had complete data. We did neither perform any transformation on the outliers, as we did not detect the presence of important outliers in our dataset that could distort our analysis. Furthermore, the distribution in the numerical variables was studied before performing any analysis with QQ plots, using non parametric tests to analyze variables that might have had outliers that could distort the results from parametric statistical methods.

## 3 Results

### 3.1 Descriptive analysis of sociodemographic and clinical data

**Table 1** shows the sociodemographic clinical characteristics of the sample. In total 115 subjects, 58 children EIPV and 57 children not EIPV. There were 30 boys in each group, the EIPV group consisted of 28 girls while 27 girls were not EIPV. The average age was 10 years, age range (8-12). 36.5% belonged to two-parent families and 35.7% had families with separated parents with maternal custody. There were not sociodemographic differences between EIPVand non-EIPV group regarding etnic, age, gender and familiar antecedents. There were differences in family structure (p=0.001), diagnosis (P=0.006) and familiar antecedents of father (p=0.015).The main ethnic groups were Caucasian making up 50.9% of EIPV children and 71% of non-EIPV children. The main DSM-5 diagnostic groups in EIPV children were disorders related to trauma and adaptive stress (43.9%) followed by affective disorders (anxiety and depression, 24.5%). In non-EIPV children, the main disorders included attention deficit (33.9%) followed by anxiety disorders (17.9%). In terms of first rank psychiatric history, 51% of parents of EIPV children presented some disorder compared to 22% in non-EIPV. In the EIPV group, 93.6% of the violence lasted more than 6 months.

**Table 1:** Sociodemographic description EIPV/Non- EIPV.

	EIPV		Non-EIPV	
	Mean	SD	Mean	SD
Age	10.36	1.25	10.11	1.35
Range (min–max)	(8-12)			
Gender	N	%	N	%
Male	30	51.7	30	52.6
Female	28	48.3	27	47.4
Total	58	100.0	57	100.0
Ethnicity				
Caucasian	29	50.9	40	71.4
Hispanic	23	40.3	11	19.6
Gypsy	1	1.8	2	3.6
Maghrebian	1	1.8	2	3.6
Others	4	7.6	2	3.6
Family structure				
Two-parent family	5	8.6	37	64.9
Single parent family	30	51.7	11	19.3
Separated parents, maternal custody	16	27.6	7	12.3
Separated parents, paternal custody	0	0	0	0
Separated parents, joint custody	8	13.8	2	3.5
Main Group Diagnoses (DSMV)				
Attention deficit hyperactivity disorder	11	19.3	19	33.9
Impulse control, and conduct disorders	3	5.3	10	17.2
Trauma and Stress Related Disorders (adjustment disorders)	26	44.7	8	14.3
Trauma and stress related disorders (PTSD and acute stress disorders)	4	7.2	2	3.6
Depressive disorders	8	14.0	2	3.6
Anxiety disorders	6	10.5	10	17.9
Others	0	0	6	10.7
Family Psychiatric History 1st rank				
Father	18	31.0	7	12.3
Mother Total	12 30	20.7 51.7	6 13	10.5 22.8
Father’s Diagnosis				
Addiction to alcohol and other toxins.	10	17	7	12,6
Affective disorders	6	10.2	0	0
Personality disorders	2	3.4	0	0
Mother’s Diagnosis				
Addiction to alcohol and other toxins	2	3.4	0	0
Affective disorders	7	11.9	4	6.8
Personality disorders	2	3.4	2	3.4
Others	1	1.7	0	0

### 3.2 Group differences in EA

Of the six factors that make up EA significant differences between the two groups were only found in the factors of differentiating emotions (DIE) and analysis of one’s own emotions (ANE). The EIPV children obtained significantly lower scores (p<0.05) on the DIE and ANE scale than the non-EIPV children (**Table 2**).

**Table 2:** Comparison of Emotional Awareness Questionnaire (EAQ) in EIPV/Non EIPV.

EAQ Factors	EIPV		Non- EIPV			
	Mean	SD	Mean	SD	P-value	Effect size
EAQ-DIE	14.67	3.65	16.29	3.18	.012*	.474
EAQ-VSE	6.00	2.06	6.40	1.90	.279	.201
EAQ-NHE	10.19	2.76	10.68	2.88	.350	.173
EAQ-BAE	9.88	2.97	9.44	3.25	.450	.141
EAQ-ANE	10.71	2.29	11.75	2.57	.024*	.427
EAQ-AOE	12.14	2.04	12.70	1.77	.117	.293

* P-value <.05.

EAQ scales: DIE, Differentiating emotions; VES, Verbal sharing of emotions; NHE, No hiding of emotions; BAE, Body awareness of emotions; ANE, Analyses of emotions; AOE, Others’ emotions. EIPV, Exposed to intimate partner violence.

### 3.3 Differences in protective factors

EIPV children also had higher scores than non-EIPV children, with a significant difference (p <0.05) on the MESSY social skills scale in loneliness, social anxiety (MESSY-SAS) and MESSY-Total. Regarding Protective Factors (PRF), children EIPV displayed significantly lower scores in autonomy (PRF-A), in the GA-RE14 resilience scale in the empathy factors (EF), internal protective factors (IPF) and Total Score of Protective Factors (PF-T), and in the self-esteem scale RSES-T (**Table 3**).

**Table 3:** Comparison of Protective Factors in EIPV/Non- EIPV Children.

Protective Factors Scales	EIPV		Non- EIPV			
	Mean	SD	Mean	SD	P-value	Effect size
PRF-A	8.069	1.275	8.556	1.148	.036*	.401
PRF-H	6.932	1.723	6.924	1.720	.979	.005
PRF-C	4.808	1.701	5.242	1.634	.172	.260
MESSY-AA	38.101	8.947	36.254	10.915	.104	.029
MESSY-ASS	34.395	8.419	31.887	7.077	.087	.323
MESSY-LSA	7.293	2.142	6.1404	1.797	.002*	.583
MESSY-F	16.681	4.384	14.769	4.284	.020*	.441
MESSY-T	107.559	18.187	99.993	19.772	.035*	.398
RSES-T	28.377	5.195	31.128	5.255	.006*	.526
GA-RE14-EF	10.955	2.119	11.973	2.230	.015*	.468
GA-RE14-EPF	11.938	2.431	12.446	2.263	.254	.216
GA-RE14-IPF	28.506	5.774	31.107	6.077	.021*	.439
GA-RE14-PF-T	51.300	8.284	55.592	8.843	.009*	.501

* P-value <.05.

AA, Aggressiveness/antisocial behavior; ASS, Appropriate social skills; LSA, Loneliness/social anxiety; F, Friendship; MESSY-TS, Total scale; RSES-T, Total self- esteem; PRF, Personal resilience factors; A, Autonomy; H, Humor; C, Creativity; GA-RE14: EF, Empathy factor; EPF, External protective factor; IPF, Internal protective factor; PF-T, Total.

### 3.4 Group differences in symptoms

**Table 4** describes the differences in symptoms between the EIPV and non-EIPV groups. Significantly higher CBCL behaviour questionnaire scores were found in EIPV children than in non-EIPV children, in Total Externalizing symptoms (CBCL-TE) and Internalizing symptoms (CBCL-TI), except for somatic complaints. Highers scores in the trait anxiety scales of the self-reported questionnaire (STAIC-AR), in total depression (DE) of the CDI self-report questionnaire, in the somatic complaints (SCL), of the mood states questionnaire (MOOD) in fear, sadness and anger were found in children EIPV. On the other hand, EIPV children obtained lower scores than non-EIPV children in state anxiety (STAIC-AE) and in happiness mood (MOOD-H).

**Table 4:** Comparison of Symptoms and Mood in EIPV/Non- EIPV.

Symptoms and Mood Scales	EIPV		Non- EIPV			
	Mean	SD	Mean	SD	P-value	Effect size
CBCL-W	5.906	3.318	4.691	3.047	.043*	.381
CBCL-AD	11.500	5.762	9.361	4.987	.036*	.397
CBCL-AP	11.314	6.180	8.622	4.826	.010*	.485
CBCL_SC	1.233	1.499	.789	1.176	.087	.130
CBCL-SP	4.500	2.683	3.479	2.414	.051	.133
CBCL-TP	4.083	2.799	2.713	1.918	.004*	.100
CBCL-DB	4.290	3.444	2.679	2.702	.003*	.228
CBCL-AB	14.936	8.109	10.758	6.841	.003*	.266
CBCL-TE	19.226	10.850	13.437	8.864	.003*	.265
CBCL-TI	18.639	9.144	14.842	8.261	.011*	.218
STAIC-SA	48.942	8.635	52.896	5.542	.025*	.139
STAIC-TA	39.755	8.942	35.636	8.229	.012*	.479
CDI-DE	15.230	6.867	10.480	6.156	.000*	.728
MOOD-F	7.67	2.122	6.63	1.686	.004*	.543
MOOD-S	7.19	2.004	5.88	1.415	.000*	.755
MOOD-H	10.224	1.654	11.268	1.307	.000*	.241
MOOD-A	8.00	1.901	6.82	1.649	.001*	.663
SCL-T	17.707	4.196	15.282	3.008	.001*	.664

* P-value <.05.

CBCL: W, Withdrawn; SC, Somatic complaints; AD, Anxious/depressed; SP, Social problems; TP, Thought problems; AP, Attention problems; DB, Delinquent behavior; AB, Aggressive behavior; TE, Total externalizing; TI, Total internalizing; STAIC: SA, State-anxiety; TA, Trait-anxiety; CDI: DE, Depression; MOOD: F, Fear; S, Sadness; H, Happiness; A, Anger; SCL: T, Total.

### 3.5 Logistic regression

Three stepwise logistic regressions were performed, including significant variables from the bivariate analyses. The first regression focused on the Emotional Awareness (EAQ) factors, with EAQ-DIE and EAQ-ANE, being the strongest predictors of group membership (EIPV/non-EIPV). The second regression, involving protective factors, included MESSY-SAS and GA-RE14-T as differentiating variables for group membership. Finally, CBCL-TE and CDI-DE symptoms were found to explain group membership.

Finally, in the last regression, conducted in three blocks, significant variables from the previous models were included. The first block consisted of the EA factors, the second block included protective factors, and the third block encompassed symptoms. The results indicated that the variables that best explained group membership between EIPV and non-EIPV were EAQ-DIE, EAQ-ANE, and CBCL-TE (see **Table 5**).

**Table 5:** Regressions of EA, symptoms, and PF in EIPV children and in non-EIPV children.

	EIPV/Non- EIPV			
	B	SD	p-val.	Exp (B)
EAQ_DE	-.144	.062	.020	.866
EAQ_AE	-.227	.090	.012	.797
CBCL-TE	.055	.022	.012	1.056

R2 de Nagelkerke = .218.

## 4 Discussion and conclusions

Regarding the first objective, the following results were obtained: DIE and ANE were significantly lower in EIPV children than in non-EIPV children, confirming the study’s first hypothesis. In relation to the second objective, EIPV children presented with more loneliness and social anxiety and more social skills problems, less resilience capacity in empathy factors (EF), internal protective factor (IPF) and Total Score of protective factors (PF-T) and less autonomy, as well as less self-esteem, therefore, this hypothesis was supported. Finally, regarding the third objective of this study, EIPV children presented with greater symptoms in Total Externalizing Symptoms and Total Internalizing Symptoms. They also displayed higher scores in the trait anxiety, in total depression, in somatic complaints, and in the mood states of fear, sadness and anger. Therefore, this hypothesis was also supported.

Finally, it was found that the variables that explain group membership in EIPV were DIE, ANE, and Total Externalizing symptoms.

### 4.1 Emotional awareness

The results on EA indicate that only DIE and ANE were significantly lower in EIPV children than in non-EIPV children. These results are in line with those obtained in some previous studies in community samples (32, 79), where it was concluded that victims of domestic violence have lower EA capacity than those who grew up in a safe environment. Katz et al. (32) found that EIPV children had more difficulties in differentiating between emotions, describing emotions, and relating emotions to their causes. Weissman et al. (79) also reported lower EA in females, but not in males, who experienced EIPV abuse during childhood and adolescence.

Acquisition of attention skills related to emotions, such as discriminating and understanding emotions, precedes attitudinal skills, which involve analyzing one’s own emotions and paying attention to the emotions of others within a social context (12). Among the six factors of EA, these two factors are considered fundamental, as they are prerequisites for a deeper analysis of the causes of emotions. Therefore, the lower capacity for DIE in EIPV children would make sense in developmental theories as an affected and stalled capacity in the face of interparental conflict. However, the lower capacity in ANE could have a different explanation, such as a psychological defense mechanism, that is, not thinking and analyzing one’s own emotions to avoid suffering when facing stressful situations such as interparental conflict.

On the other hand, several authors have demonstrated that in households where violence is present, parents tend to have difficulty engaging in conversations where emotions are expressed honestly and openly with their children, as well as providing less support, acceptance, and affection (42, 80). This type of interaction with little attention to children’s emotions could also be impacting the low capacity to analyze one’s own emotions due to a lack of learning to understand one’s emotional world.

These findings also align with previous findings on child maltreatment by (81), who observed that children who are victims of child maltreatment tend to have disorganized, vague, and negative internal representations, as well as difficulties labelling and identifying emotions as well as recognizing facial expressions (26–28).

Interestingly, the children in this sample did not show differences in VSE, NHE, BAE an AOE factors of EA, that is, the ability to speak about emotions with others, in the tendency to express one’s own emotions frankly and openly, in the ability to understand the nexus between emotional activation and body symptoms and attending to the emotions of others. A possible explanation for the lack of significant differences could be related to methodological issues. For instance, the type of scale used to measure these factors of EA may not be sufficiently sensitive to capture such differences, or, being a self-report measure, children might underestimate or overestimate their levels of these factors of EA. Measuring EA could be improved by using alternative approaches that focus on the ability to recognize emotions at different levels of complexity, employing everyday emotional situations based on relational scenarios that an individual might experience, and assessing the individual’s capacity to identify and differentiate the emotions associated with those situations. Additionally, it is important to consider that we are referring to two groups of children with underlying psychopathology, which may also affect their EA.

However, when exploring somatic complaints with SCL, and the empathy factor scale (EF), it was observed that EIPV children had more somatic complaints and lower levels of empathy. This suggests that a deeper exploration of these specific competencies within EA could reveal their impact on children EIPV.

### 4.2 Protective factors

The results indicate that EIPV children have a lower capacity for empathy compared to children who have not been EIPV. These findings align with studies by Graham-Bermann (82) that highlight deficits in EIPV minors’ ability to connect with others and interpret social cues, making it difficult for them to empathize with the perspectives and feelings of others.

The results also indicate that the EIPV children presented lower social skills, lower competence in establishing friendships and greater social isolation compared to the non-EIPV group. These findings are consistent with previous research within the theoretical framework of developmental theories, which has shown that EIPV children tend to exhibit less developed social skills, communication problems, and challenges in interpreting non-verbal cues. Consequently, they face difficulties in interpreting social language, which in turn affects their satisfaction and success in interpersonal relationships (83). Minors in this situation usually exhibit antagonistic behaviors in the social sphere that endanger their social relationships and integration (43, 83). It could also be explained by attachment theory (7). Children EIPV present with increased anxiety and fear or with avoidance or aggressive participation in reaction to parents behaviour that may be reproduced with peers.

In the same way, Kath et al. (84), reported that children from homes with higher levels of domestic violence experienced greater difficulty in forming close friendships at the age of 9. Furthermore, Davies et al. (44) conducted a longitudinal study and found that increased parental conflict during early school years predicted higher levels of emotional insecurity and difficulties in forming affiliative relationships with friends during adolescence, as well as poorer social competence.

Furthermore, the EIPV group exhibited lower self-esteem compared to the non-EIPV group, supporting prior research indicating an association between domestic violence and low self-esteem (85). Aligned with findings previously reported in children who had experienced other types of abuse, self-esteem and social competence are considered mediating variables in the adjustment mechanisms of children in high-risk situations (86, 87).

On the other hand, the results provide further evidence that children experiencing EIPV perceive less support from their family, which also aligns with previous studies that demonstrated how affection and negative behaviors resulting from conflict transfer to parent-child interactions, leading to a reduction in positive interactions between parents and children (41, 42).

Finally, the descriptive results highlight that children EIPV are more likely to have parents with mental disorders than those not exposed to IPV. This emphasizes the importance of parental mental health in understanding the lack of parental involvement in children’s emotional development in IPV contexts. These findings are consistent with Levendosky et al. (88), who discussed the connection between partner violence and parental mental health, suggesting that parental mental disorders may lead to partner violence and negatively affect parental involvement in their children’s emotional growth. Wolfe et al. (89) also examined how parental mental health affects children experiencing partner violence, reinforcing this theory.

### 4.3 Symptoms

Regarding the symptoms, significant differences were found between the groups, with EIPV children exhibiting more internalizing and externalizing symptoms, trait anxiety, total depression, somatic complaints, and in the mood states of fear, sadness, and anger, compared to non-EIPV children.

These results are in line with previous studies. It is widely documented (43, 44) that children and adolescents who have been EIPV present with worse mental health, affective problems, and behavioural and cognitive symptoms compared with the unexposed. Likewise, several authors (90, 91) found that children who had suffered emotional abuse had more problems of inattention, impulsivity, and hyperactivity associated with attention deficit hyperactivity disorder (ADHD). Moreover, poor EA is positively associated with externalizing disorder diagnoses in children with ADHD. Factor et al. (92) concluded that externalizing problems are indicative of dysregulated emotional reactivity rather than planned misbehaviour.

Previous studies have found that difficulties in EA are positively correlated with depressive and anxiety symptoms (12, 93, 94). The experience of prolonged and frequent negative mood states can affect EA, as it makes it harder to detect differences in emotions (93). Along the same line, a metanalysis found that children with more anxiety have difficulty with emotional competencies such as acceptance, understanding, expression of emotions, as well as emotion regulation (95).

Children EIPV exhibited a higher number of somatic complaints than their non-EIPV counterparts. These findings are consistent with previous studies on EIPV children who frequently experienced physical health problems, particularly related to eating, sleep, pain issues, and self-harm (48). It is known that in response to stress or negative affect, humans exhibit physiological responses such as increased heart rate, perspiration, and muscle tension, which are typical and aid in coping (96). Furthermore, several psychological variables known to augment negative affect are associated with an increase in somatic complaints. For instance, poor management of emotional states can lead to heightened somatization and an increase in somatic complaints (15).

Regarding moods, children EIPV exhibited heightened levels of fear, sadness, and anger. The predominance of these emotions, typically regarded as negative, is understandable given that these children are subject to stressful family environments. This exposure correlates with their increased difficulties in peer relationships and a diminished sense of self-worth.

Finally, the descriptive findings revealed that children EIPV were diagnosed with Trauma and Stress Related Disorders at a substantially higher rate compared to children not EIPV. These results could support studies indicating that children subjected to IPV undergo multiple traumatic events over an extended period, closely mirroring the characteristics of CPTSD (49, 50). Furthermore, Frewen et al. (30) determined through a systematic review that alexithymia symptoms might be linked to developmental trauma exposure, independent from PTSD symptoms.

### 4.4 Limitations and future directions

The study encountered several limitations that need to be acknowledged. Among these, there was a limitation in the time needed to achieve the complete sample due to the challenging process of obtaining consent from both parents in cases involving parental violence because, in cases where there was high conflict between the parents, we had to provide separate information about the study and clarify any doubts that arose regarding the use of the data and confidentiality. Moreover, conducting the study during the COVID-19 pandemic and the associated lockdown measures in Spain created difficulties in maintaining contact with families. To address the mentioned limitation and continue data collection during confinement, it was necessary to adapt the recruitment method through online and telephone communications. The information sheets and questionnaires were adapted to the REDCap software to be administered online during the lockdown period. This adaptation allowed the study to proceed despite the physical restrictions imposed by the COVID-19 pandemic.

Besides these limitations, we emphasize that this project had an exploratory purpose, and no correction of the p-values was applied. The results of this paper should be further analyzed in projects that specifically study some of the aspects we have considered. Additionally, data on EA were collected through self-report measures which come with limitations, such as interpretation difficulties in children, not only in the sample of EIPV children but also in the children with other mental disorders. These limitations may affect the accuracy and reliability of the data collected.

Finally, socioeconomic status and other potential confounding factors were not collected. Recognizing these gaps, we suggest that future studies could benefit from including such variables to further strengthen the research outcomes.

The study acknowledges the potential for selection bias as it recruits participants from mental health centers. This may limit how representative the sample is of all children exposed to IPV, especially those not seeking mental health services. This limitation underlines the necessity for future research to employ broader recruitment strategies to enhance the generalizability of the results like community-based sampling, community organizations working with families and children, such as social services or collaborations with schools, including primary and secondary schools.

The greatest strength of this study is that it is a clinically relevant study since there are no previous studies that compare EA, protective factors and symptoms, specifically in a clinical sample of EIPV and non-EIPV children. Additionally, unlike previous studies that focus on overall EA, this study analyzed the six specific factors that constitute EA, providing a more refined understanding of their relevance among EIPV and non-EIPV children. These results are highly relevant because they compare EA in children EIPV and non-EIPV children within a clinical sample, where the capacity of EA in non-EIPV children is likely already compromised due to general clinical vulnerability. Also, these results will be helpful in improving diagnostic precision, considering the symptoms that underlie the major trauma-associated mental disorders, according to DSM-5 criteria, in children EIPV, along the lines of cross-sectional diagnosis. Including a structured assessment of EA as well as both internal and external protective factors in children aged 8 to 12 exposed to IPV would enable the implementation of a psychological intervention aimed at reducing symptoms and improving the mental well-being of children, while also facilitating the acquisition of protective factors to foster resilience, with the aim of indirectly reducing symptoms.

The findings can be applied in specific interventions, such as parenting therapies to teach parents how to develop emotional skills and improve emotional communication. Additionally, they can be developed in specific group therapies to enhance emotional skills in patients with alexithymia or emotional dysregulation issues. Finally, it would be useful to implement school-based interventions to improve social skills.

Future studies may need to further explore how EA acts as a mediator for protective factors and for externalizing and internalizing symptoms in children EIPV. It is also important to continue examining EA in EIPV adolescents in relation to borderline personality symptoms, as well as eating disorders. Concurrently, research should aim at developing and tailoring effective therapeutic interventions that focus on enhancing EA, such as emotional and interpersonal regulation therapy. Furthermore, it would be necessary to explore how specific therapies, such as Emotion Regulation and Interpersonal Therapy (TREI), Mindfulness, and Dialectical Behavioral Therapy (DBT), which focus on emotional awareness, affects it development. Additionally, longitudinal studies should be conducted to assess the development of EA during adolescence.

### 4.5 Conclusions

We can conclude that this study provides valuable information on the involvement of EA and protective factors in EIPV children in a clinical sample, it also provides a clear difference with children with mental disorders but without EIPV, which makes the results even more powerful. The results show there was lower EA, fewer protective factors (both external and internal) related to resilience and more internalizing and externalizing symptoms as well as somatic complaints, in EIPV children than in non-EIPV children.

## Data Availability

The raw data supporting the conclusions of this article will be made available by the authors, without undue reservation.
